# Atypical Manifestations of Cutaneous Leishmaniasis in a Region Endemic for *Leishmania braziliensis*: Clinical, Immunological and Parasitological Aspects

**DOI:** 10.1371/journal.pntd.0005100

**Published:** 2016-12-01

**Authors:** Luiz Henrique Guimarães, Adriano Queiroz, Juliana A. Silva, Silvana C. Silva, Viviane Magalhães, Ednaldo L. Lago, Paulo Roberto L. Machado, Olívia Bacellar, Mary E. Wilson, Stephen M. Beverley, Edgar M. Carvalho, Albert Schriefer

**Affiliations:** 1 Serviço de Imunologia, Hospital Universitário Professor Edgard Santos, Universidade Federal da Bahia, Salvador, Brazil; 2 Instituto Nacional de Ciência e Tecnologia em Doenças Tropicais (INCT-DT), Salvador, Brazil; 3 Centro de Formação em Saúde, Universidade Federal do Sul da Bahia, Teixeira de Freitas, Brazil; 4 Department of Internal Medicine, University of Iowa, Iowa City, Iowa, United States of America; 5 Department of Microbiology, University of Iowa, Iowa City, Iowa, United States of America; 6 VA Medical Center, Iowa City, Iowa, United States of America; 7 Department of Molecular Microbiology, Washington University School of Medicine, St. Louis, Missouri, United States of America; 8 Centro de Pesquisas Gonçalo Moniz, Salvador, Brazil; 9 Departamento de Ciências da Biointeração, Instituto de Ciências da Saúde, Universidade Federal da Bahia, Salvador, Brazil; University of Notre Dame, UNITED STATES

## Abstract

**Background:**

Atypical cutaneous leishmaniasis (ACL) has become progressively more frequent in Corte de Pedra, Northeast Brazil. Herein we characterize clinical presentation, antimony response, cytokine production and parasite strains prevailing in ACL.

**Methodology/Principal Findings:**

Between 2005 and 2012, 51 ACL (cases) and 51 temporally matched cutaneous leishmaniasis (CL) subjects (controls) were enrolled and followed over time in Corte de Pedra. Clinical and therapeutic data were recorded for all subjects. Cytokine secretion by patients’ peripheral blood mononuclear cells (PBMC) stimulated with soluble parasite antigen in vitro, and genotypes in a 600 base-pair locus in chromosome 28 (CHR28/425451) of the infecting *L*. *(V*.*) braziliensis* were compared between the two groups. ACL presented significantly more lesions in head and neck, and higher rate of antimony failure than CL. Cytosine–Adenine substitutions at CHR28/425451 positions 254 and 321 were highly associated with ACL (p<0.0001). In vitro stimulated ACL PBMCs produced lower levels of IFN-γ (p = 0.0002) and TNF (p <0.0001), and higher levels of IL-10 (p = 0.0006) and IL-17 (p = 0.0008) than CL PBMCs.

**Conclusions/Significance:**

ACL found in Northeast Brazil is caused by distinct genotypes of *L*. *(V*.*) braziliensis* and presents a cytokine profile that departs from that in classical CL patients. We think that differences in antigenic contents among parasites may be in part responsible for the variation in cytokine responses and possibly immunopathology between CL and ACL.

## Author Summary

*Leishmania braziliensis* is the main cause leishmaniasis that affects skin and upper airways mucosa in Brazil. Atypical cutaneous leishmaniasis (ACL) presentations are difficult to diagnose and treat, and do not fit the classical descriptions of the disease. It is not clear whether ACL share unique features or which are its determinants. In our research we found that ACL occur in the vicinity of the more common localized cutaneous leishmaniasis (CL) patients in the study region, so individuals are equally exposed to risks of acquiring either disease. We also found that *L*. *braziliensis* from ACL share genetic traits not commonly identified among parasites from CL, so parasite strain is one plausible risk factor for ACL. Finally, blood cells from ACL produced levels of immune proteins known to participate in the pathogenesis of leishmaniasis that were distinct from those of CL patients. We conclude that atypical cutaneous leishmaniasis in Northeast Brazil may be considered a clinical presentation per se, in part resulting from infection of human beings with distinct strains of *L*. *braziliensis*. Precise identification of ACL is important because it usually does not respond to drugs commonly used to treat leishmaniasis in Brazil, but readily responds to other treatment options available.

## Introduction

Tegumentary leishmaniasis is caused by protozoa of the genus *Leishmania* and presents a worldwide incidence of 0.7 to 1.2 million cases per year. Brazil, Colombia, Peru, Costa Rica, Algeria, North Sudan, Ethiopia, Syria, Iran and Afghanistan account for approximately 75% of the global incidence of disease [1]. In the New World, *Leishmania (Viannia) braziliensis* is the predominant species causing American tegumentary leishmaniasis (ATL) [2]. There are four distinct forms of ATL recognized in Brazil: localized cutaneous leishmaniasis (CL), mucosal leishmaniasis (ML), disseminated leishmaniasis (DL) and diffuse cutaneous leishmaniasis (DCL).

The clinical, pathologic and immunologic features of the different types of ATL are distinct [3]. CL is the most prevalent form and is characterized by one or few ulcers with elevated borders, occurring mainly in exposed areas of the patients' bodies, like upper limbs, lower limbs, and face [3]. CL often presents with markedly enlarged lymph nodes, once termed “bubonic leishmaniasis” [4]. Mildly sore lymphadenopathy usually develops early during infection before cutaneous lesion fully develops. It affects locally draining nodes, and recedes during treatment, frequently preceding ulcer healing.

ML affects primarily the nasal mucosa, and is documented in approximately 3% of patients with history of CL [5, 6]. In the past, ML accounted for up to 24% of ATL in the region of Corte de Pedra [7]. The remarkable drop in its incidence may have been caused by a combination of factors like improvement of local population´s access to early diagnosis and treatment of ATL, or changes in the human and / or parasite populations in the area. DL presents with more than ten, and sometimes with several hundred acneiform, papular and ulcerated lesions spread onto at least two non-contiguous areas of the patients' body surfaces [8–10]. DCL is characterized by infiltrated, nodular and non-ulcerated lesions often affecting face, limbs and trunk of patients [11].

Several reports have called attention to atypical cutaneous leishmaniasis (ACL), which consists in a form of ATL that does not fit into any of the four disease definitions above. Authors have referred to ACL cases as sporotrichoid [12], erysipeloid [13], recidiva cutis [14] or zosteriform [15]. We previously described the clinical features of ACL in an area hyperendemic for *L*. *(V*.*) braziliensis* transmission in the Northeast of Brazil. In that study, ACL accounted for 1.9% of all ATL diagnosed at the leishmaniasis clinic that serves as reference for diagnosis and treatment of the disease in the region of Corte de Pedra [16].

Unusual clinical presentations of leishmaniasis have been attributed to a range of possible causes including host immunosuppression, co-morbidities and pregnancy, as well as environment factors and strain of the parasite [12–15, 17]. The majority of such patients do not present with clinical co-morbidities or HIV infection, and are not using immunosuppressive drugs [18]. Thus other explanations must be sought to address the diversity of disease forms.

Studies of parasite genomic DNA have led us to conclude that the *L*. *(V*.*) braziliensis* population in Corte de Pedra is complex, and that different parasite strains are associated with distinct clinical presentations of disease [19, 20]. More recently, we described a locus starting at position 425,451 on chromosome 28 of *L*. *(V*.*) braziliensis* (locus CHR28/425451) that is polymorphic among strains of the parasite in the region [19]. We found that certain haplotypes of single-nucleotide polymorphisms (SNP) and insertions-deletions (indel) in CHR28/425451 are associated with increased risk ratios of DL in that sample [19]. Of note, DL itself was considered an atypical manifestation of ATL in the past, but it has steadily increased in prevalence over the decades, reaching a consistent fraction of 4.0% of all ATL in Corte de Pedra [8–10].

In the current study we evaluated the association between SNPs found in the locus CHR28/425451 of *L*. *(V*.*) braziliensis* and disease outcome, to address whether differences in parasite strain may be one determinant leading to ACL in Corte de Pedra. We also evaluated whether such patients might share a common immune profile, comparing the secretion of a panel of cytokines between immune cells of ACL and CL stimulated with *L*. *(V*.*) braziliensis* antigen in vitro. Our data showed that ACL found in the northeast of Brazil is caused by genotypically distinct strains of *L*. *(V*.*) braziliensis* and presents a cytokine profile that departs from that found in classical CL.

## Methods

### Study area

Corte de Pedra is composed of 20 municipalities in a rural area located in the southeastern region of the state of Bahia, in the northeast of Brazil. Corte de Pedra falls within the geographic coordinates (latitude / longitude) 14°/39°, 13°/39°, 14°/40°, 13°/40°. *Lutzomyia (Nyssomyia) whitmany* and *Lu*. *(N*.*) intermedia* are the main vectors transmitting *L*. *(V*.*) braziliensis* in Corte de Pedra. The leishmaniasis clinic of the Health Post Dr. Jackson Costa serves as reference for the diagnosis and treatment of ATL in the region. Residents of this area work mostly in agriculture, which is often carried out in primary or secondary forests.

### Patients

Fifty-one patients diagnosed with ACL in the leishmaniasis clinic at the Health Post between January 2005 and July 2012 were included in the study. This corresponded to the total ACL diagnosed in the study period. An equal number of CL subjects were randomly recruited, matched for the date of initial evaluation at the Health Post leishmaniasis clinic, to serve as control group. ACL was defined by the presence of unusual cutaneous crusted, lupoid, sporotrichoid, vegetative, verrucous, or zoster-like lesions due to *L*. *(V*.*) braziliensis*. CL was defined as a single skin ulcer without patients' upper airway or digestive mucosal involvement. The initial screening diagnosis of ATL was based on parasite isolation in culture of lesion specimens and / or a positive leishmania skin test combined with compatible histopathological findings. All suspected ATL cases had the diagnosis confirmed and *Leishmania* species determined by qPCR, using DNA from tissue fragments of lesions.

Relevant clinical data such as the presence of co-morbidities or the use of immunosuppressive drugs that could affect the immune responses and ATL outcomes were investigated in all patients. Blood tests to determine levels of glucose, urea nitrogen and hepatic enzymes were performed, as well as serological tests for HIV, hepatitis B virus (HBV), hepatitis C virus (HCV) and human T-cell lymphotropic virus type 1 (HTLV-1).

### ATL treatment

Patients were treated with intravenous pentavalent antimony (Glucantime) at a dose of 20mg/kg/day for 20 days for individuals with CL, and 30 days for those with ACL. Patients refractory to antimony were further treated with Glucantime plus Pentoxifylline (400mg, 3 times daily for 20 days), or with amphotericin B (0.5 mg / kg body weight, 3 times per week until reaching a total dose of 1.0 g to 1.5 g). Failure of antimony therapy was defined as the persistence of active lesions after two full courses with Glucantime.

### Mapping CL and ACL in Corte de Pedra

High-resolution distribution of CL and ACL cases was determined by acquisition of geographic coordinates using the GPS device Garmin GSX 60 (Garmin, Riverton, WY, USA). Because *L*. *(V*.*) braziliensis* is believed to be transmitted mostly within plantations where residents of the region live and work, patient residences were used as reference points for standardization purposes. Collected data were statistically compared as described below, and plotted for visual inspection onto a high-definition satellite photograph of Corte de Pedra (ENGESAT, Curitiba, Brazil), using ArcGis version 10 (Environmental Systems Research Institute Inc., Redlands, CA, USA).

### Parasites

The *L*. *(V*.*) braziliensis* isolates used in the present study were cultured from aspirates of the borders of skin lesions. Aspirated material was immediately suspended in biphasic liver infusion tryptose/Novy, McNeal, Nicolle (LIT/NNN) medium and incubated at 26°C for one to two weeks. The suspension was then transferred to complete Schneider’s medium supplemented with 10% heat-inactivated fetal calf serum and Gentamicin 50 mg/mL (Sigma-Aldrich), and incubated at 26°C for up to another two weeks. We were able to successfully isolate *L*. *(V*.*) braziliensis* from 16 ACL and 38 CL patients. Parasites were frozen without further subculture in 10% DMSO, 90% growth medium in liquid nitrogen, and thawed prior to DNA extraction for species and genotype determination.

### *L*. *(V*.*) braziliensis* genomic DNA extraction and parasite species determination by PCR

Genomic DNA was extracted from suspensions containing approximately 10^6^ promastigotes as previously described [19, 20] then re-suspended in 100μL of TE (Tris-HCl 10mM, EDTA 1mM pH 8.0) buffer. Long-term storage DNA aliquots were kept at –70°C, while test samples were maintained at –20°C until used. *Leishmania* species was determined by a serial real-time quantitative PCR assay system [21].

### Genotyping *L*. *(V*.*) braziliensis* from CL and ACL patients

Parasites were genotyped according to the haplotypes of polymorphic nucleotides in the locus CHR28/425451, previously shown to distinguish *L*. *(V*.*) braziliensis* strains in Corte de Pedra [19]. Primers 5´:TAAGGTGAACAAGAAGAATC and 5´:CTGCTCGCTTGCTTTC were used to amplify a 622 nucleotide-long segment in CHR28/425451 from parasite genomic DNA as previously described [19]. Amplicons were cloned using the Original TA Cloning Kit pCR 2.1 VECTOR (Invitrogen, Thermo Fisher Scientific Co., MA, USA), according to manufacturer’s instructions. Briefly, the amplicons were inserted by overnight ligation into PCR 2.1 plasmids, which were used for chemical transformation of competent DH5α *Escherichia coli*. Plasmid minipreps were generated from four recombinant bacteria colonies per study isolate [22]. Amplicon cloning was confirmed by digestion analysis, using Eco RI restriction endonuclease (Invitrogen).

Plasmid inserts were sequenced by the Sanger method with primers complementary to the M13 vector sequences. Sequencing was performed at Macrogen Inc. (Seoul, South Korea). Mega 5.0 software [23] was used to align the sequences with the CHR28/425451 clones obtained from the panel of *L*. *(V*.*) braziliensis* parasites, in order to determine the SNP/indel haplotypes detectable in each study isolate.

### Peripheral blood mononuclear cells (PBMC) separation and cytokine production upon stimulation with *L*. *(V*.*) braziliensis* antigen in vitro

Blood collection for cytokine testing was performed at diagnosis before treatment of enrolled patients was initiated. PBMC from 20 ACL and 20 CL patients were isolated by density gradient centrifugation with Ficoll-Hypaque (Sigma, St. Louis, MO, USA). The cells were cultured in RPMI 1640 medium supplemented with 5% fetal calf serum, 100 U penicillin/mL and 100μg streptomycin/mL (GIBCO BRL, Grand Island, NY, USA). Briefly 3 × 10^6^ cells/mL were plated in 24-well flat bottom microtiter plates (Falcon, Becton Dickinson, Lincoln Park, NJ, USA) and kept with media alone (unstimulated), or were stimulated with 5 μg/mL of soluble leishmania antigen (SLA). SLA was prepared from a stock *L*. *(V*.*) braziliensis* isolate derived from a CL patient of Corte de Pedra. Cell cultures were incubated at 37°C and 5% CO2 for 72 hours, or 96 hours in the case of IL-17 determination. IFN-γ, TNF, IL-10 and IL-17 levels were determined in supernatants by ELISA (BD Bioscience Pharmingen, San Jose, CA, USA). The results were expressed in pg/mL.

### Statistical analyses

Comparison of clinical data between CL and ACL patients employed Fisher’s exact and Mann Whitney tests in GraphPad Prism 5.0 (GraphPad Software, Inc., San Diego, CA, USA). Univariate linear regressions were fitted to evaluate the association between disease duration and both therapeutic failure and cytokines production. Then the association between these variables was controlled by clinical presentation in a multivariate model. Statistical analyses were performed using STATA software (version 14.1; StatCorp, College Station, TX, USA). P Values < 0.05 were considered statically significant.

We compared the geographic distribution of CL and ACL in Corte de Pedra using the Cuzick and Edward’s test in the geostatistical package Clusterseer version 2.2.4 (Terraseer Inc., Ann Arbor, MI, USA). This test detects significance when two groups of geographic events distribute differently over the study area. For evaluating the association between *L*. *(V*.*) braziliensis* strain and ACL, the distribution frequencies of SNPs and corresponding haplotypes in CHR28/425451 were compared between CL and ACL cases by Fisher`s exact test, using GraphPad Prism 5.0 (GraphPad Software, Inc.). All comparisons were considered significant at p<0.05.

### Ethical considerations

This study was approved by the Institutional Review Board of the Federal University of Bahia (document of approval: CAAE– 3041.0.000.054.07). Written consent was obtained from all participating subjects. All patients whose photographs are shown gave their permission to publish the pictures after the photographing author (LHG) identified himself and explained the purpose of the photograph.

## Results

### Demographic and clinical features of ACL and CL Patients

**Fig 1:**
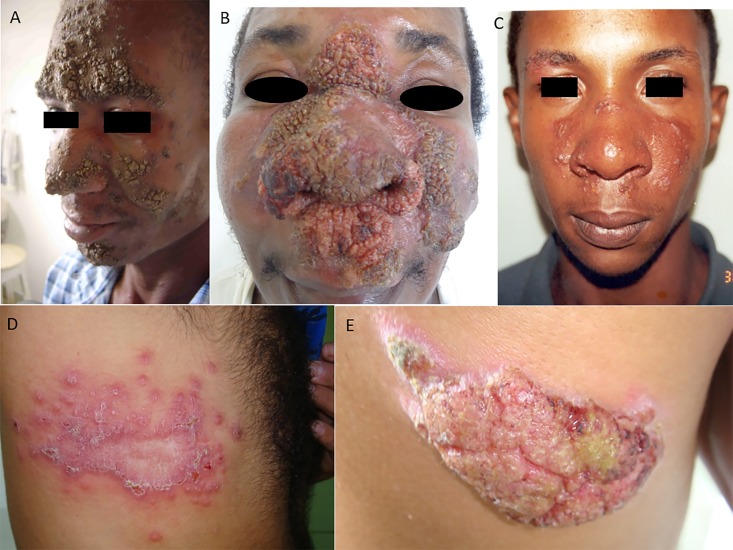
Examples of common presentations found among atypical cutaneous leishmaniasis (ACL) patients in Corte de Pedra region, Northeast Brazil. (A) Crusted, (B) verrucous, (C) lupoid, (D) Zoster-like and (E) vegetative lesion. Pictures depict actual ACL study subjects. All patients whose photographs are shown gave their permission to publish the pictures after the photographing author (LHG) identified himself and explained the purpose of the photograph.

All patients' biochemical parameters were within normal ranges, and viral serology was negative. No patients were using immunosuppressive drugs. Age, gender, clinical manifestations and response to antimony therapy in study subjects are shown in Table 1. The age and gender distribution was similar in the 2 groups. Patients with ACL had significantly more lesions, particularly above the waist, and in the head and neck regions than those with CL. Atypical lesions could be classified as vegetative (N = 19), crusted (N = 14), vegetative ulcer (N = 10), verrucous (N = 3), lupoid (N = 2), sporotrichoid (N = 2) and zoster-like (N = 1) (Fig 1). Disease duration was significantly longer among ACL individuals (Table 1). Approximately sixty-three percent of CL patients healed with one course, and ninety-eight percent with up to two courses of antimony (Table 1). In contrast, only approximately thirty-five percent of ACL patients responded to treatment, including those requiring two courses with Glucantime (Table 1).

**Table 1:** Clinical variables compared between 51 atypical (ACL) and 51 localized cutaneous leishmaniasis (CL) temporally matched patients from Corte de Pedra, Brazil.

	ACL	CL	P
Age [average ± SD]	30.6± 14.4	28.8±9.6	0.68^*^
Male [N (%)]	38 (74.5)	36(70.6)	0.83^#^
Illness duration [mean ± SD]	123 ± 249.5	37.2±26.2	0.0006^*^
Number of lesions [mean ± SD]	41 ± 139	1±0.6	0.0001^*^
Lesion above waist [N (%)]	43 (84.3)	19(37.3)	<0.0001^#^
Lesion in head and/or neck [N (%)]	26 (51)	5(9.8)	<0.0001^#^
Failure to first antimony treatment [N (%)]	40 (78.4)	19 (37.3)	0.0001^#^
Failure to two or more antimony treatments [N (%)]	34 (85.0)	1(5.3)	<0.0001^#^

*Mann-Whitney test.

#Fisher's exact test.

The association between disease duration and both therapeutic failure and cytokines production (please see cytokine profiles section below) were controlled by clinical presentation in a multivariate model. No significant association was observed.

### *L*. *(V*.*) braziliensis* strains drawn from ACL differ from those obtained of geographically close CL patients

**Fig 2:**
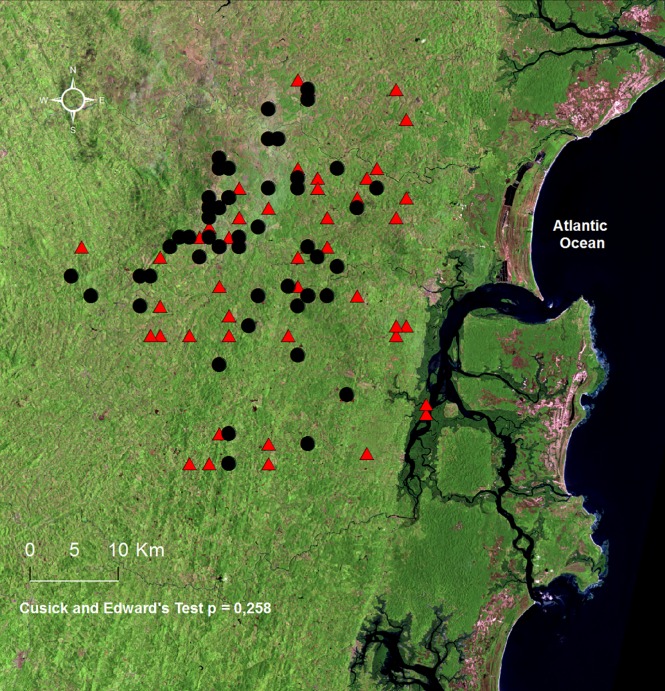
Atypical (ACL) and localized cutaneous leishmaniasis (CL) cases distribute similarly in Corte de Pedra, Brazil. ACL (N = 51) and temporally matched CL (N = 51) cases diagnosed between 2005 and 2012 in Corte de Pedra were mapped, and the resulting sets of geographic events were statistically compared. Black dots and red triangles correspond to ACL and CL patients, respectively. Total number of dots and triangles plotted is smaller than the actual number of corresponding cases due to overlap of some patients' geographic coordinates. The two manifestations of leishmaniasis present similar distributions in Corte de Pedra, with Cuzick and Edward´s comparison yielding non-significant (p = 0.26). For details refer to Methods section.

Geographic coordinates of the residences of ACL and CL patients revealed that both groups of subjects were widely spread over the affected region (Fig 2), presenting statistically similar distributions in Corte de Pedra (Cusick and Edward's p = 0.258). This suggests the subjects may be equally exposed to environmental variables that may influence the outcome of ATL.

All patients were infected with *L*. *(V*.*) braziliensis*. In order to evaluate whether different parasite strains may constitute one risk factor for development of ACL, we compared SNPs at polymorphic positions in locus CHR28/425451 among *L*. *(V*.*) braziliensis* successfully isolated from 16 ACL and 38 CL patients. These SNPs defined six haplotypes, according to the nucleotides found at positions 30, 254, 286 and 321 within CHR28/425451 (Table 2). Haplotypes CCCA, TATA, CACA and TCCA could only be found in parasites from ACL patients, occurring in 10 (62.5%) of these subjects (Table 2). Furthermore, A alleles at positions 254 and 321were highly associated with ACL and could not be detected in parasites drawn from CL patients (Table 3). These findings indicate that certain strains of *L*. *(V*.*) braziliensis* are more frequent among patients with atypical lesions.

**Table 2:** Comparison of SNP haplotypes frequencies in locus CHR28/425451 between *L*. *(V*.*) braziliensis* isolated from 16 atypical (ACL) and 38 localized cutaneous leishmaniasis (CL) temporally matched patients from Corte de Pedra, Brazil.

Haplotype* (positions are 30,254,286,321)	CL	ACL	P#	Odds ratio	CI 95%
CCCC	32	10	0.148	0.31	0.08–1.2
TCTC	26	10	0.76	0.77	0.23–2.6
CCCA	0	2	0.084	13.3	0.6–293.7
TATA	0	3	0.008	31.4	1.53–644.2
CACA	0	4	0.006	27.72	1.39–552.0
TCCA	0	1	0.29	7.45	0.287–193.2

* Haplotypes are based on SNP contents at positions 30, 254, 286 and 321 within the locus starting at position 425,451 on the parasite´s chromosome 28.

^**#**^Fisher's exact test.

**Table 3:** Comparison of SNP allele frequencies in locus CHR28/425451* between *L. (V.) braziliensis* isolated from 16 atypical (ACL) and 38 localized cutaneous leishmaniasis (CL) temporally matched patients from Corte de Pedra, Brazil. SNPs in this table correspond to individual SNPs in haplotypes delineated in Table 2.

Position in CHR28/425451 (SNP)	CL	ACL	P^#^	Odds ratio	CI 95%
30 (C–T)	26	11	1.0	1.02	0.29–3.58
254 (C–A)	0	7	<0.0001	95.53	5.03–1814
286 (C–T)	26	10	0,76	0.77	0.227–2.61
321 (C–A)	0	10	<0.0001	124.4	6.47–2393

^**#**^Fisher's exact test.

* Locus starts at position 425,451 on the parasite´s chromosome 28.

### Cytokine profiles in ACL and CL subjects

**Fig 3:**
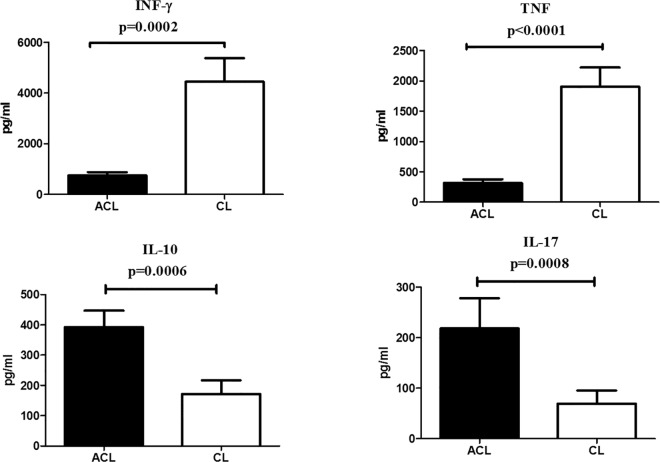
Cytokine levels in supernatants of peripheral blood monuclear cells from atypical (ACL) and localized cutaneous leishmaniasis (CL) patients stimulated with *L*. *(V*.*) braziliensis* soluble antigen (SLA) in vitro. Levels of IFN-γ, TNF and IL-10 were determined by ELISA in culture supernatants of PBMCs stimulated in vitro with SLA for 72 h. Levels of IL-17 were determined after 96 h of stimulation. Data are expressed in pg/mL. Black and white columns depict average cytokine levels produced by ACL and CL patients, respectively. Bars denote standard error of the means. All comparisons rendered significant to p<0.001 by Mann Whitney test.

Given the notorious role of immune response in the outcomes of ATL, we investigated whether ACL patients might present an immune profile distinct from that of CL. Exploring a limited panel of key cytokines, we compared the production of IFN- γ, TNF, IL-10 and IL-17 between PBMCs from ACL and CL cases, stimulated in vitro with *L*. *(V*.*) braziliensis* antigen (Fig 3). ACL derived PBMCs consistently produced lower levels of IFN-γ (p = 0.0002) and TNF (p<0.0001), but higher levels of IL-10 (p = 0.0006) and IL-17 (p = 0.0008) than CL patient PBMCs. Of note, the limited dispersion in cytokine data, particularly for IFN-γ and TNF, suggests that ACL represents a fairly homogeneous group of individuals, despite the various possible skin presentations of their disease.

## Discussion

Variants of disease caused by *L*. *(V*.*) braziliensis* present diagnostic and therapeutic challenges even to health care professionals working in regions endemic for CL. Several cases reports of atypical *L*. *(V*.*) braziliensis* infections have been related to impairment of the immune response [16], use of immunosuppressive drugs [17], malnutrition or pregnancy [16, 24]. However, the majority of ACL do not seem to be related to obvious risk factors [16]. In the current study we showed that *L*. *(V*.*) braziliensis* strain and levels of inflammatory cytokines are associated with atypical manifestations of CL.

Subjects with ACL presented to health professionals after a longer duration of illness than individuals with CL. In this endemic region where self-reporting is common, we hypothesize that this was due to lack of self-recognition of the disease because of the atypical presentation. Typical CL ulcers are usually recognized by people living in Corte de Pedra, causing most patients to voluntarily seek care within 30 days of its onset. It is also likely that some of these individuals reach the reference leishmaniasis clinic at the Health Post after having been seen at several other facilities in the region, without effective diagnosis of their illness. Also noteworthy, ACL lesions were more frequent above the waist and in the face, but such patients did not present with mucosal involvement. This is in contrast to CL, in which lesions above waist and in the face indicate patients are at a high risk of developing ML [16].

Antimony is the first line drug for CL treatment in Brazil. It is distributed freely by the Brazilian Ministry of Health. However, failure of antimony has been on the rise in the country. Whereas in the early 80`s only 10% of individuals with CL failed antimony therapy in Brazil [25], this figure has increased and now ranges from 44 to 53% [26]. A second course of treatment with antimony usually cures close to 100% of initially refractory CL patients. In marked contrast, we observed that more than 60% of ACL failed two courses with antimony (Glucantime).

The main rescue treatment in cases of antimony failure was amphotericin B. Only 5 ACL cases underwent antimony plus pentoxifylline therapy. These patients tolerated well the treatment, and none of them had to interrupt pentoxifylline use. The only side effect reported was mild nausea.

In the Old World, genetic diversity among *L*. *major* strains has been associated with differences in the clinical presentation of leishmaniasis [27]. We have previously reported the complexity of *L*. *(V*.*) braziliensis* population in Corte de Pedra, and association between this complexity and the clinical manifestations of ATL [19, 20]. In those reports we found that DL was associated with genetically distinct strains of *L*. *(V*.*) braziliensis* [19, 20]. DL has been rapidly emerging in Corte de Pedra [10, 28], and like ACL it does not respond well to antimony therapy. Originally, DL was considered an atypical manifestation of ATL, although due to increasing frequency, DL can no longer be considered “atypical”. The genotypic differences identified between isolates in the current study serve to expand our previous documentation that strain aligns with clinical disease form, indicating that parasite strain may also be a partial determinant of ACL. It is important to note that alleles found among parasites of ACL cases could not be found in the CHR28/425451 locus of *L*. *(V*.*) braziliensis* from DL patients diagnosed in the region during the study period.

A number of *Leishmania* genetic loci have been implicated in resistance to antimonial compounds [29, 30]. Recently published field data have indicated that *Leishmania (Viannia)* species naturally infected with *Leishmania* RNA virus 1 (LRV1) are associated with increased disease pathology [31–33], and antimony treatment failure in ATL patients of Andean countries in South America [34]. However, published and preliminary data have not as yet reported the presence LRV1 within *L*. *(V*.*) braziliensis* strains from eastern Brasil, including the Corte de Pedra region [35]. Thus, antimonial treatment failures in ACL may arise by mechanisms other than the presence of LRV1. These interesting questions will be addressed in future studies.

The ultimate goal of developing molecular markers of parasite isolates is to eventually utilize these markers to predict clinical disease outcome, and apply this information for diagnostic and therapeutic management of ATL. Toward this goal, we examined genotypes of *L*. *(V*.*) braziliensis* isolates at positions 254 and 321 in CHR28/425451, and found that they segregated isolates according to form of human disease. The CA haplotype was found only among isolates from patients with CL, whereas AC was specific for parasites isolated from cases of ACL. It is possible that diagnostic tools based on PCR-defined genotypes might be developed to detect whether an ATL patient has been infected with genotype marking a strain that usually associates with either ACL or CL, thus putting them at risk for a specific type of disease manifestation [16]. ACL has proven significantly more refractory to antimony than CL, but fully responsive to amphotericin B. Thus such information might become of importance for choice of therapy. In this respect, ACL patients might benefit most if they were preferentially treated with either amphotericin B or with the combination of antimony and pentoxifylline, avoiding initial therapy with antimony alone.

Classical CL is characterized by a strong type 1 T cell response to *Leishmania* antigen, with secretion of high levels of IFN-γ and TNF [36]. Although excessive IFN-γ and cytotoxic CD8^+^ T cells may contribute to the inflammatory response that leads to ulcer development [37–39], the importance of IFN-γ as a defense mechanism in CL is well established. Suppressed production of IFN-γ is associated with systemic spread of *L*. *infantum* and *L*. *donovani* [40], and low IFN-γ is observed in DCL patients infected with *L*. *amazonensis* [11]. In several disease forms, the microbicidal effects of IFN-γ are antagonized by IL-10 in vivo [36] or in vitro in macrophages [41, 42]. The cytokine responses detected in PBMCs from ACL patients in the current study were characterized by lower IFN-γ and higher IL-10 than in PBMCs from subjects with CL. We hypothesize that this imbalance may favor parasite growth, and may partially explain the exacerbated pathogenesis of ACL as well as the high rate of therapeutic failure.

The role of IL-17 in the pathogenesis of leishmaniasis has not been completely elucidated. IL-17 is produced by peripheral blood cells and in lesions of CL patients [43], and IL-17 has been correlated with pathology in ML [44]. Since IL-17 is negatively regulated by IFN-γ [45], we can only speculate that the low production of IFN-γ we observed in ACL patients may be favoring higher IL-17 production, which may be related to an increased tissue inflammation and the atypical phenotypes of lesions.

We have previously reported that strains of *L*. *(V*.*) braziliensis* that cause DL are genotypically different from those that cause CL [19]. Herein we further expand that observation to document distinct genotypes associated with isolates from individuals with ACL. Additionally, we also found that the profile of key cytokines produced by PBMCs from ACL subjects was substantially different from those reported for all other manifestations of *L*. *(V*.*) braziliensis* infection. These findings may contribute to our understanding of the pathogenesis of ACL, and ultimately to a more logical approach to management of this and other unusual forms of ATL.

## Supporting Information

S1 Checklist STROBE Checklist. (DOC)
